# Panner’s Disease: The Vacuum Phenomenon Revisited

**DOI:** 10.5334/jbsr.1647

**Published:** 2018-10-12

**Authors:** Aliaksandr Anisau, Magdalena Posadzy, Filip Vanhoenacker

**Affiliations:** 1 AZ Sint-Maarten Mechelen/Duffel, BE; 2 W. Dega Orthopaedic and Rehabilitation University Hospital of Karol Marcinkowski University of Medical Sciences Poznan, PL; 3 AZ Sint-Maarten and University (Hospital) Antwerp/Ghent, BE

**Keywords:** Panner, osteochondrosis, capitellum, Cone Beam CT, vacuum phenomenon

A seven-year-old boy presented with pain in his left elbow with a subtle swelling following a fall. There was a limited range of motion, mostly an extension deficit of the elbow. Conventional radiography (CR) of the left elbow (Figure 1) revealed joint effusion (asterisk) and irregular delineation of the articular contour of the capitellum with a radiolucent line in the subchondral bone (black arrows). There was also faint sclerosis of the capitellum (white arrows). Subsequent cone beam computed tomography (CT) (Figure 2) depicted a crescent-shaped subchondral vacuum phenomenon in the capitellum (black arrows). The diagnosis of Panner’s disease was made and the patient was treated conservatively, with rest, temporary immobilization and subsequent remobilization. The clinical follow-up was uneventful.

**Figure 1:**
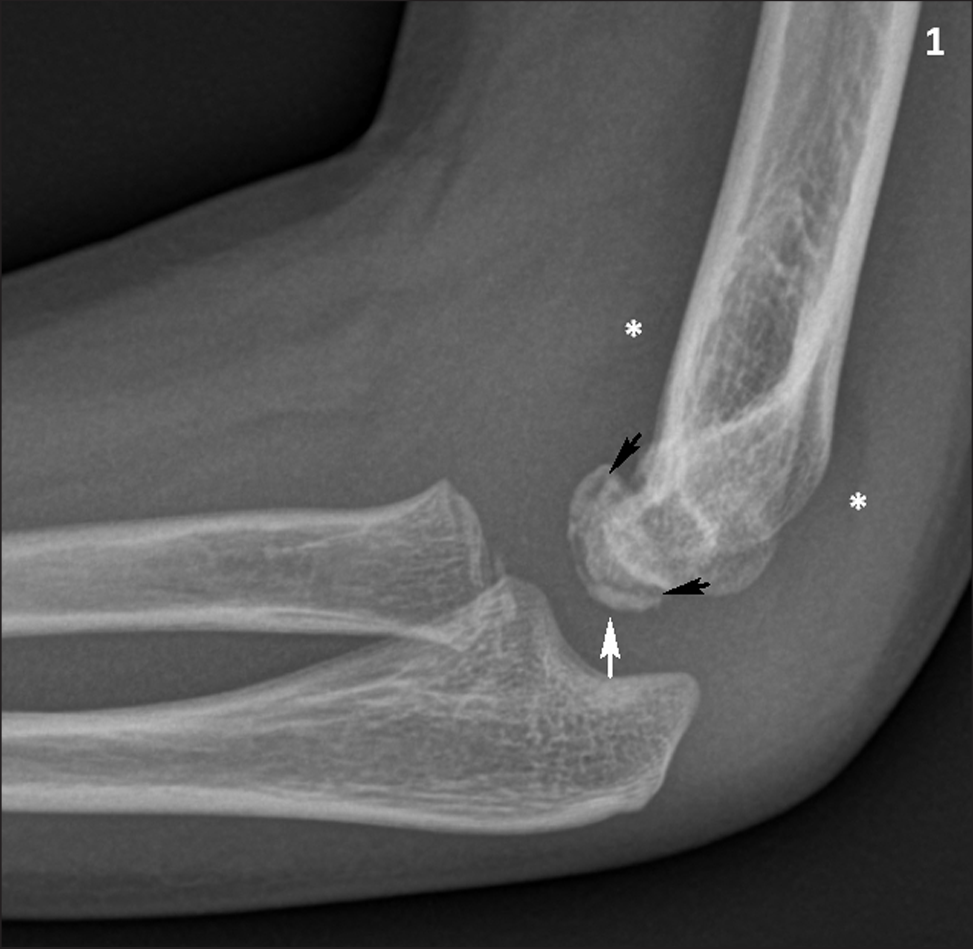
Conventional radiography of the right elbow, lateral view. The capitellum has a slightly irregular articular contour (white arrow) and there is a radiolucent line in the subchondral bone (black arrows). Note slight joint effusion with displacement of the elbow fad pads (asterisks).

**Figure 2:**
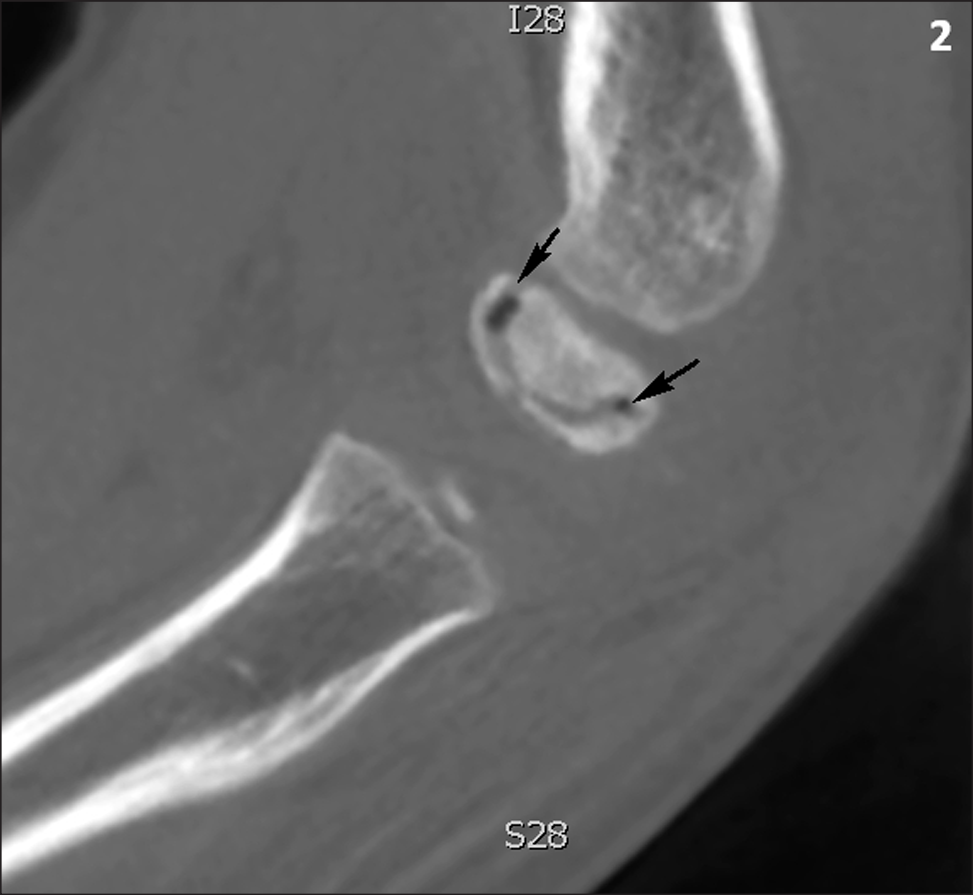
Cone beam CT of the right elbow, sagittal reformatted image. The capitellum has an increased density. There is a subchondral crescent-shaped vacuum phenomenon (black arrows).

## Comment

Panner’s disease represents an osteochondrosis of the ossification centre of the capitellum. The disease is most prevalent in boys in the first decade of life. Often there is history of trauma or repetitive stress, more specifically valgus stress or increased axial load to the elbow. Valgus stress is probably the most important factor in the pathogenesis of Panner’s disease under the age of five years [1].

Pathophysiologically, the condition results from disordered endochondral ossification of the capitellum, which may be caused by ischemia due to disruption of the vascular supply of the capitellum by repetitive stress [1]. An acute traumatic event can aggravate an already existing but subclinical Panner’s disease.

Clinical findings include pain, swelling, stiffness and a limited range of motion. Typically there is extension deficit of approximately 20° whereas flexion deficit is less frequent [1]. The symptoms are exacerbated by activity and mitigated by rest [1].

Radiographical findings include changes in morphology, contour deformity, collapse and increased density of the capitellum [1]. Detection of a subchondral vacuum phenomenon is a relatively rare finding, but highly diagnostic.

Cone beam CT is more sensitive to depict subtle changes than CT, in particular the vacuum phenomenon, at the expense of still a relatively low radiation dose. The vacuum phenomenon, which represents gas accumulation within the subchondral cleft, is a sign of bone ischemia and may indicate avascular necrosis and impending articular collapse, similar to the Legg – Calvé – Perthes disease. When reparation and re-ossification eventually occurs, the vacuum-phenomenon disappears.

Magnetic resonance (MR) imaging features of Panner’s disease include signal inhomogeneity in the ossification centre of the capitellum, bone marrow oedema and elbow effusion [1]. As intraosseous gas is less conspicuous on MR, this technique is less suited for detecting the subchondral vacuum phenomenon.

The main differential diagnosis of Panner’s disease is osteochondritis dissecans, which occurs in older children and adolescents in the second decade of life. Typically, the cartilage of the capitellum is intact in Panner’s disease and therefore the overall prognosis is favourable. In osteochondritis dissecans, articular cartilage damage may occur requiring a prolonged immobilization and resulting in an often poorer outcome.

Treatment of Panner’s disease is generally conservative, consisting of rest and sometimes immobilization, restriction of sport activities. Most lesions heal without sequelae [1].
